# Observation of Shape, Configuration, and Density of Au Nanoparticles on Various GaAs Surfaces via Deposition Amount, Annealing Temperature, and Dwelling Time

**DOI:** 10.1186/s11671-015-0950-z

**Published:** 2015-05-27

**Authors:** Daewoo Lee, Ming-Yu Li, Mao Sui, Quanzhen Zhang, Puran Pandey, Eun-Soo Kim, Jihoon Lee

**Affiliations:** College of Electronics and Information, Kwangwoon University, Nowon-gu, Seoul 139-701 South Korea; Institute of Nanoscale Science and Engineering, University of Arkansas, Fayetteville, AR 72701 USA

**Keywords:** Au nanoparticle, Catalyst, Self-assembly, Volmer–Weber growth model

## Abstract

Metallic nanoparticles have been widely witnessed in many applications: serving as the catalysts for various nanowire systems, as the active mediums of various device applications, and also for the nanoscale templates for hybrid quantum structures. In the performance of devices and configurations of the resulting nanostructures, the size and density of nanoparticles play critical roles. In this paper, the control of self-assembled Au droplets on GaAs (100), (110), and (111) is systematically investigated through the variation of deposition amount (DA), annealing temperature (AT), and dwelling time (DT). Based on the Volmer–Weber growth model, the formation of Au droplets and dramatic evolution of Au nanostructures on various GaAs surfaces is observed from the Au clusters to the round-dome shapes with the AT variation between 250 and 550 °C. With the systematic DA control, a radical size and density evolution of Au droplets shows the size expansion of over 400 % in average height and 800 % in average lateral diameter, while the density shows over two orders of decrease. With the DT variation, the self-assembled Au droplets tend to grow larger due to the Ostwald ripening while a clear distinction among the surface indexes is observed.

**Electronic supplementary material:**

The online version of this article (doi:10.1186/s11671-015-0950-z) contains supplementary material, which is available to authorized users.

## Background

**Fig. 1:**
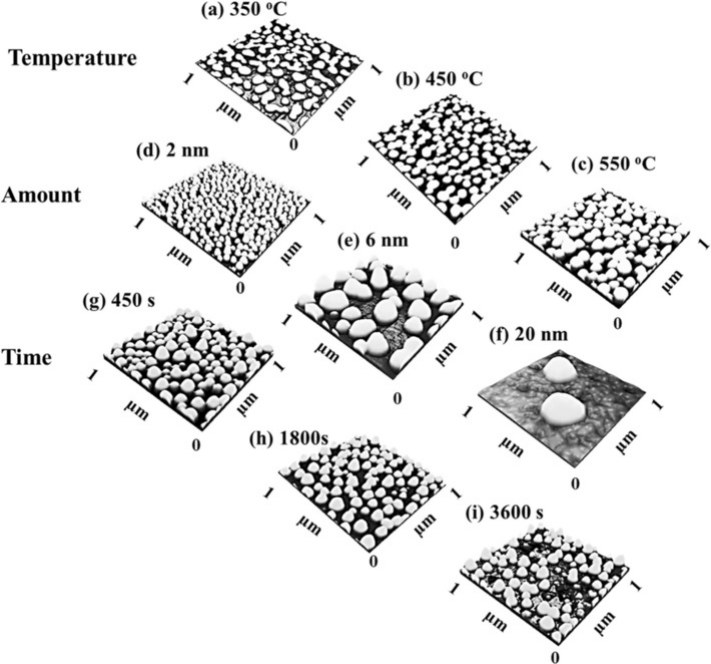
Illustration of the fabrication of self-assembled Au droplets by the control of annealing temperature, deposition amount, and dwelling time on GaAs (111). **a**–**c** Atomic force microscope (AFM) side views showing the surface morphologies of Au droplets at 350, 450, and 550 °C. **d**–**f** AFM side views of Au droplets along with the deposition amount variation as labeled. **g**–**i** Au droplet formation with the variation of dwelling time. **a**–**i** Images are 1 × 1 μm^2^

Metallic nanoparticles have drawn a lot of attention in various fields for the device applications [1–10], hybrid nanostructures [11–20], and catalysts of nanowires (NWs) [21–29]. For instance, the size and density of metallic nanoparticles can directly determine the electron mobility, sensitivity, and localized surface plasmon resonance frequency in field-effect transistors [1, 2], solar cells [3, 4], and various sensors [5–10], thus offering new approaches and even opportunities to optimize the device performances. Metallic nanoparticles can also be utilized for in situ surface nanohole drilling without the introduction of crystal damages and defect formation via the droplet-etching technique [11–13], and thus can provide the nanoscale templates for various hybrid quantum nanostructures including the localized quantum dot molecules [14–16], quantum rings [17–19], and NWs [20] based on the droplet epitaxy (D-E). In addition, metallic nanoparticles, especially Au droplets, have been widely reported as the catalyst for various NWs such as Si, Ge, GaAs, InP, InAs, ZnO, and GaN through the vapor-liquid–solid (VLS) mechanism [21] in various epitaxial approaches such as metal-organic chemical vapor deposition [22], pulsed laser deposition [23, 24], chemical beam epitaxy [25], and molecular beam epitaxy [26]. Depending on the surface index utilized and growth conditions, the resulting NWs can be formed in various cross-sections such as triangle, squares, trapezoid, and hexagon and even the quality of NWs such as the formation of the stacking faults (SF) can be significantly affected [27–29]. Furthermore, Au particles can find various applications in biology and nanotechnologies [30–36]. Up to now, however, the control of Au droplets by the systematic variation of the annealing temperature (AT), deposition amount (DA), and dwelling time (DT) on various GaAs surfaces is somewhat lacking; thus, in this paper, the control of self-assembled Au droplets on GaAs (111), (110), and (100) is systematically investigated through the variation of DA, AT, and DT. Figure 1 shows an overview of the AT, DA, and DT variation on GaAs (111) as an example, and in order to clearly investigate each effect, only one parameter was varied for each series. As clearly seen in Fig. 1a–c, depending on the AT, the configuration of Au nanostructures evolve dramatically from the irregular wiggly shape in Fig. 1a to the round-dome-shaped droplets in Fig. 1c, and the Au droplet formation on GaAs can be described based on the Volmer–Weber growth model [37, 38]. Meanwhile, the Au droplets quite sensitively respond to the DA variation as clearly seen in Figs. 1d–f, and very dense Au droplets to super-large droplets can be relatively simply controlled, which can be described by a simply thermodynamic diffusion theory. With the DT variation, the size increase of the Au droplets was associated with the density decrease and was equally observed on various GaAs surfaces, which can be described by the Ostwald ripening [39–41]. The results are systematically analyzed with respect to the atomic force microscopy (AFM), energy-dispersive X-ray spectrometry (EDS) spectra, Fourier filter transform (FFT) power spectra, cross-sectional line-profiles and root-mean-square (RMS) roughness, as well as the droplet dimension and density summary, respectively.

## Methods

### General Materials and Substrates

In this study, the effects of DA, AT, and DT were systematically investigated on GaAs (111), (110), and (100), common GaAs indices apart from the high indices, which can represent all possible lower indices in the zinc blend structure. Generally, the (001) surface with a square lattice terminated with Ga or As is polar. The (110) with a rectangular lattice is nonpolar, which is caused by the equal Ga and As atom distribution at the surface. The (111) surface-terminated Ga(A) or As(B) has a hexagonal polar structure [42, 43]. The GaAs substrates were epi-ready with an off-axis of ±0.1 ° from the American Xtal Technology (AXT, Inc.). Additional file 1: Figure S1 shows the surface morphologies of bare (a) GaAs (100), (b) (110), and (c) (111) surfaces. Additional file 1: Figure S2 shows the photoluminescence spectra of the GaAs (100), (110), and (111), and Additional file 1: Figure S3 shows the Raman spectra of GaAs (100), (110), and (111) substrates, respectively.

### Fabrication of Self-Assembled Au Droplets

Initially, samples were bonded on an Inconel holder by indium and treated with a degassing process at 350 °C for 30 min under 1 × 10^−4^ Torr in a pulsed laser deposition (PLD) chamber. To study the DA effect on various GaAs surfaces, the DA variation between 2 and 20 were carried out at a uniform growth rate of 0.5 Å/s with an ionization current of 3 mA under 1 × 10^−1^ Torr in an ion-coater chamber. Additional file 1: Figure S4 shows the surface morphologies with (a) 2, (b) 4, (c) 6, and (d) 20 nm thickness of Au deposition on GaAs (111) as examples. Subsequently, the substrate temperature (*T*_sub_) was automatically ramped up to 550 °C at a ramping rate of 1.83 °C/s, and the annealing process was systematically controlled by a computer-operated recipe in a PLD chamber under 1 × 10^−4^ Torr. The samples were uniformly annealed for 450 s at the target *T*_sub_, and to ensure the uniformity of resulting Au droplets, the *T*_sub_ was immediately quenched down to an ambient temperature in order to prevent the Ostwald ripening [39–41]. To study the AT effect, the AT variation between 250 and 550 °C were carried out, and to maintain the consistency, the DA and DT of 2.5 nm and 450 s were used uniformly. Similarly, the DT variation was performed for 150, 450, 900, 1800, and 3600 s at the target *T*_sub_ of 550 °C after an equal DA of 2.5 nm.

### Characterization of Au Droplets

Subsequent to the sample fabrications, surface morphologies were characterized by an AFM with a non-contact mode. The Si tip had a radius of less than 10 nm curvature with a length ~125 μm. The spring constant of the tip was ~40 N/m, and the resonant frequency was ~170 kHz (NSC16/AIBS, μmasch). The cantilever of the tip was coated with ~30 nm Al on the backside to enhance reflection of laser to improve the sensitivity of scanning. In order to minimize the tip effects for lateral size measurement, the AFM characterization was performed with the same type of tips from a single batch. The image processing, 2-D FFT power spectrum, and line-profiles were methodically analyzed by using XEI software (Park Systems). The 2-D FFT power spectrum is acquired by converting height distribution from a spatial domain to a frequency domain using FFT and thus represents the intensities of height frequencies and directionality. Additionally, elemental characterization was performed by an EDS system in vacuum with the spectral mode (Thermo Fisher Noran System 7).

## Results and Discussion

**Fig. 2:**
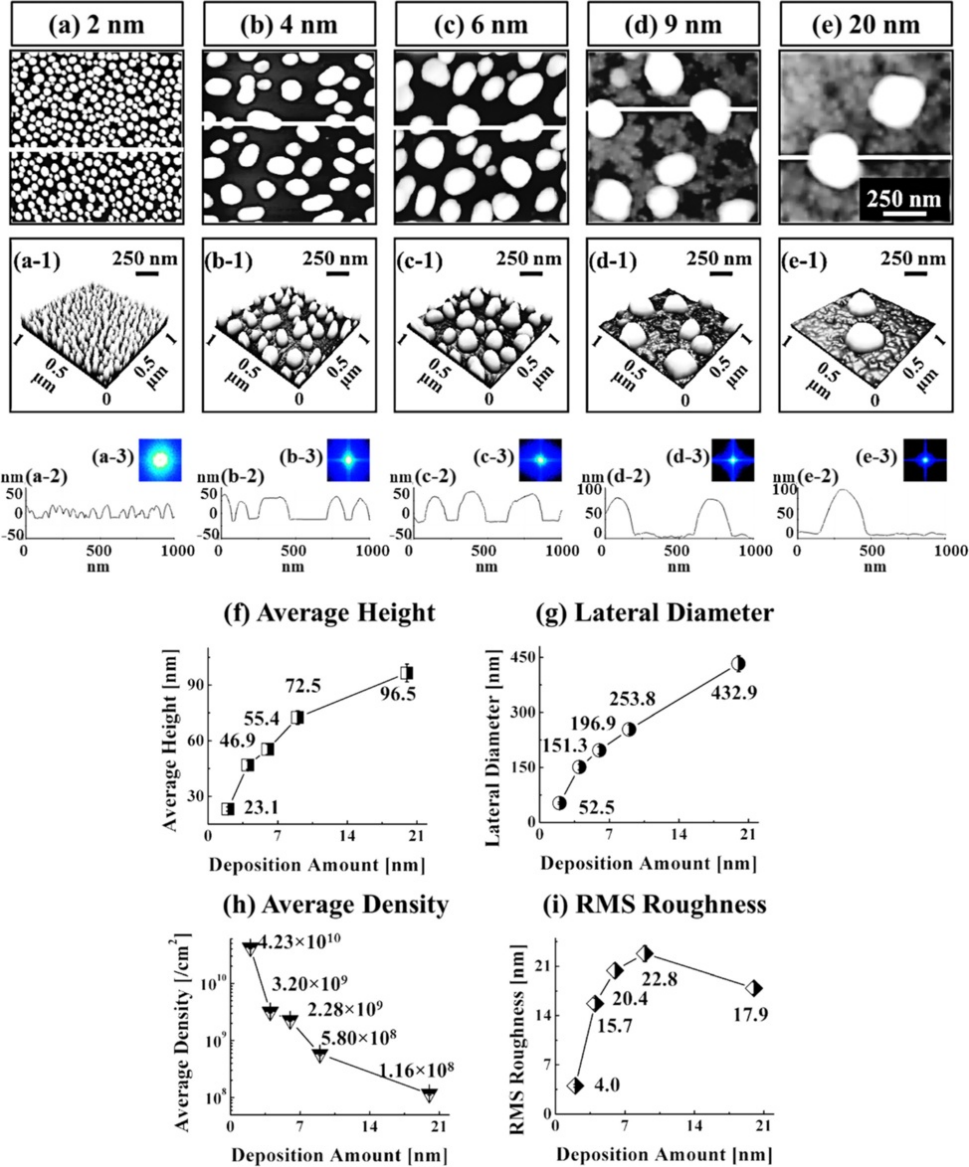
Self-assembled Au droplets synthesized via the variation of deposition amount (2–20 nm) on GaAs (111). The resulting Au droplets are shown with AFM top views in (**a**–**e**). AFM images are 1 × 1 μm^2^. Cross-sectional line-profiles in (*a-1*)–(*e-1*) are acquired from the *white lines* in (**a**–**e**). 2-D Fourier filter transform (FFT) power spectra are shown in (*a-2*)–(*e-2*). **f**–**i** Average height, lateral diameter, density, and root-mean-squared (RMS) surface roughness. *Error bars* are ±5 %

**Fig. 3:**
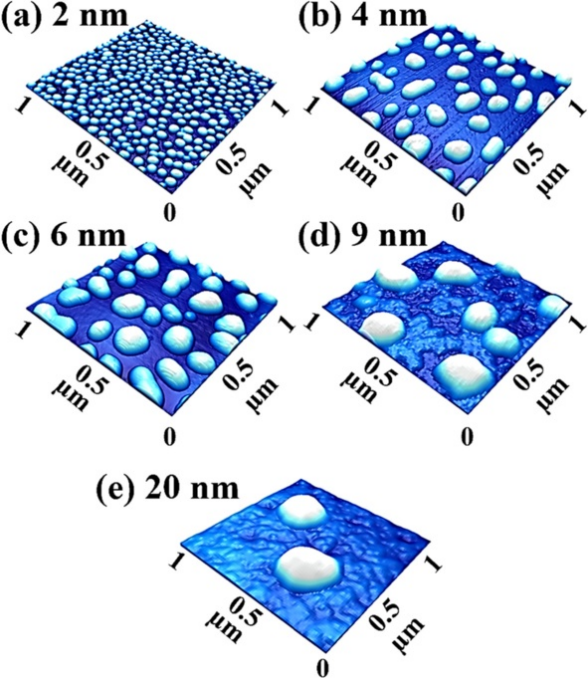
Three dimensional (3-D) AFM side views of the self-assembled Au droplets controlled via the variation of deposition amounts (2–20 nm) on GaAs (111). The self-assembled Au droplets were fabricated under the fixed annealing temperature (AT) at 550 °C for the annealing time of 450 s. **a**–**e** AFM side views of 1 × 1 μm^2^

**Fig. 4:**
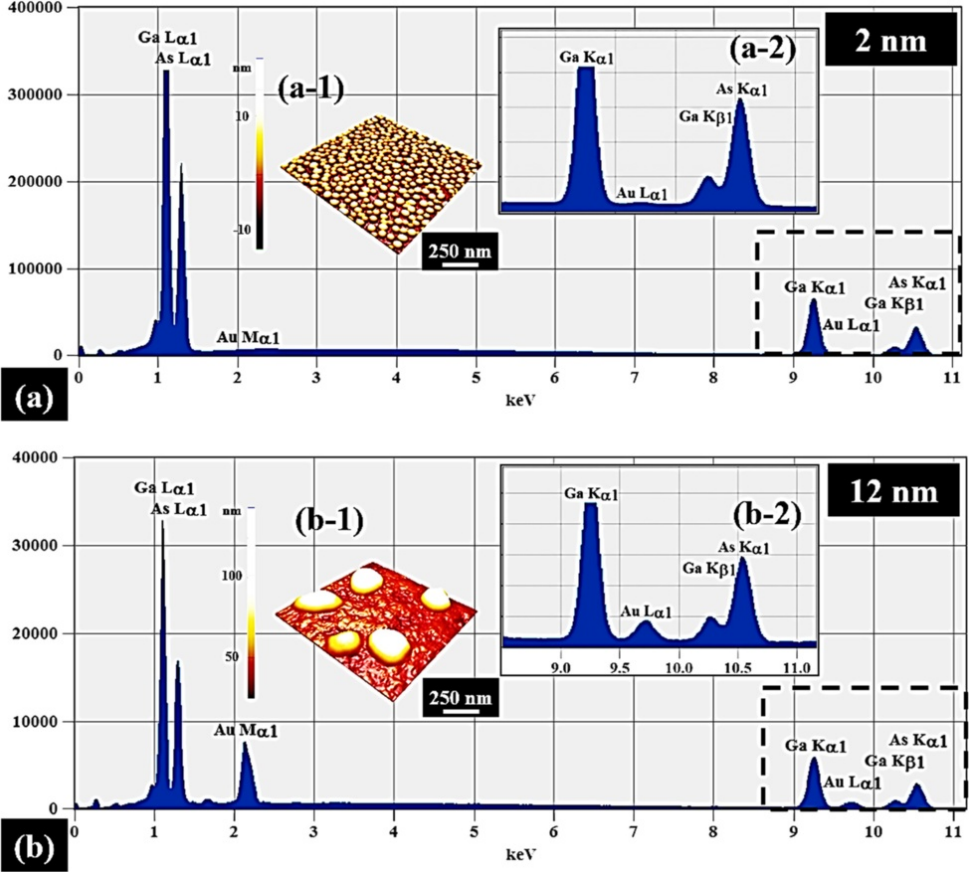
Energy-dispersive X-ray spectroscopy (EDS) spectra with 2 nm deposition in (**a**) and 12 nm in (**b**) on GaAs (111). *a-1* and *b-1* corresponding AFM side views of 1 × 1 μm^2^. *a-2* and *b-2* enlarged spectra between 9 and 11 KeV

Figure 2 shows the self-assembled Au droplets synthesized by the DA variation between 2 and 20 nm on GaAs (111). Fig. 3 shows the three dimensional (3-D) AFM side views of the corresponding samples. As clearly seen in the AFM images, only a slight change in the DA led to a significant size expansion as well as a drastic density decrement of the self-assembled Au droplets within the DA range as shown in Figs. 2a–e and 3a–e, which suggests that the Au droplets are quite very sensitive to the DA modification. The formation of self-assembled Au droplets on GaAs surfaces can be described based on the Volmer–Weber (V-W) growth model [37, 38], namely, E_I_ > E_S_, the stronger binding energy between Au adatoms (E_I_ = ~3.01 eV) than that between the substrate and Au adatoms (E_S_ = ~2.96 eV) [44]. Being provided with the sufficient thermal energy for surface diffusion, Au adatoms can diffuse to form nucleus. Once nuclei are formed on random lower energy sites, they can absorb nearby surrounding adatoms to form 3-D islands as the surface energy is lower at the nucleus sites. Given that additional materials are provided with the increased DAs within the diffusion length, the Au adatoms can keep being absorbed to form larger 3-D islands to further lower the surface energy, which in turn can result in the extended dimension of Au droplets to minimize the surface energy further. This growth mode of nanoscale metal droplets can also be observed on other substrates such as polystyrene, poly methyl methacrylate [37], and TiO_2_ [38]. Fig. 2f–h summarizes the average height (AH), lateral diameter (LD), and average density (AD) of the resulting Au droplets as a function of DA. With the 2 nm DA, very densely packed round-dome shaped Au droplets were fabricated on GaAs (111) as shown in Figs. 2a and 3a with an AH of 23.1 nm, a LD of 52.5 nm, and an AD of 4.23 × 10^10^/cm^2^. With the 4 nm DA, the self-assembled Au droplets grew larger as described and showed a slight elongation with a lower density as presented in Figs. 2b and 3b. The AH was sharply increased to 46.9 nm by × 2.03 as in Fig. 2f, and the LD was increased to 151.3 nm by × 2.88 as in Fig. 2g. Accordingly, the AD was drastically dropped to 3.20 × 10^9^/cm^2^ as in Fig. 2h, which is a decrease by × 13.22 as compared to the 2 nm DA. The increased DA can lead to an increased size of Au droplets as described, and the size increase can lower the surface energy, and thus, the chemical potential can be further increased. The absorption of adatoms thus can be further increased due to the enlarged absorption boundary. Likewise, with the 6 and 9 nm DAs, the self-assembled Au droplets kept growing larger while the average density was further reduced as revealed in Figs. 2c–d and 3c–d. Specifically, the AH increased to 55.4 and 72.5 nm and the LD was increased to 196.9 and 253.8 nm while the AD was decreased to 2.28 × 10^9^ and 5.80 × 10^8^/cm^2^, respectively. Finally, with the 20 nm DA, very large Au droplets were synthesized with a much reduced density as shown in Figs. 2e and 3e: the AH, 96.5 nm; LD, 432.9 nm; and AD: 1.16 × 10^8^/cm^2^. The size and density evolution was also clearly evidenced by the FFT power spectra: initially, the over 80 % of bright spot with 2 nm DA was gradually decreased along with the increased DAs due to the reduced height frequencies, which is quite consistent with the density decrease. In terms of the RMS surface roughness, it was constantly increased up to 9 nm with the value of 22.8 nm due to the dominance of the dimensional increase, but after then, it began to be decreased likely due to the dominance of the density decrease over the dimension increase. Figure 4 shows the EDS spectra of the self-assembled Au droplets synthesized with the 2 and 12 nm DAs, and Fig. 4a-2 and b-2 shows the enlarged spectra between 9 and 11 KeV. As shown, in comparison between two spectra, the two appear to be quite similar in overall peak positions. The Kα1 of Ga and Kβ1 of As peaks are present at 9.243 and 10.532 KeV, and similar the Lα1 of Ga and Lβ1 of As peaks are present at 1.096 and 1.282 KeV with quite similar intensities, which are from the substrates. However, the presentence of the Au Mα1 peak at 2.123 KeV with 12 nm DA in Fig. 4b appeared very clear while the Mα1 peak with 2 nm DA was hard to identify. In the same way, the Au Lα1 peak with 12 nm DA was also much more noticeable than the one with 2 nm as revealed in Fig. 4a-2 and b-2. The difference in Au peaks should have been induced by the DA difference and the interaction volume of the X-ray with the Au atoms. In brief, for the DA variation, the results suggest that the size and density of the self-assembled Au droplets are quite sensitive to DA variation, resulting in the gradual explosion of dimensions in the AH, and LD associated with the decreased AD over two orders of range and quite similar behaviors were observed on the other two substrates of GaAs (110) and (100). On the contrary, the evolution of Au droplets on soft polymeric substrates, such as polystyrene (PS) and poly methyl methacrylate (PMMA), was reported [37] to progress based on the coalescence model, in which Au film formation was observed as a function of DA variation after the four distinctive stages of nucleation, lateral growth, coalescence, and the vertical growth.

**Fig. 5:**
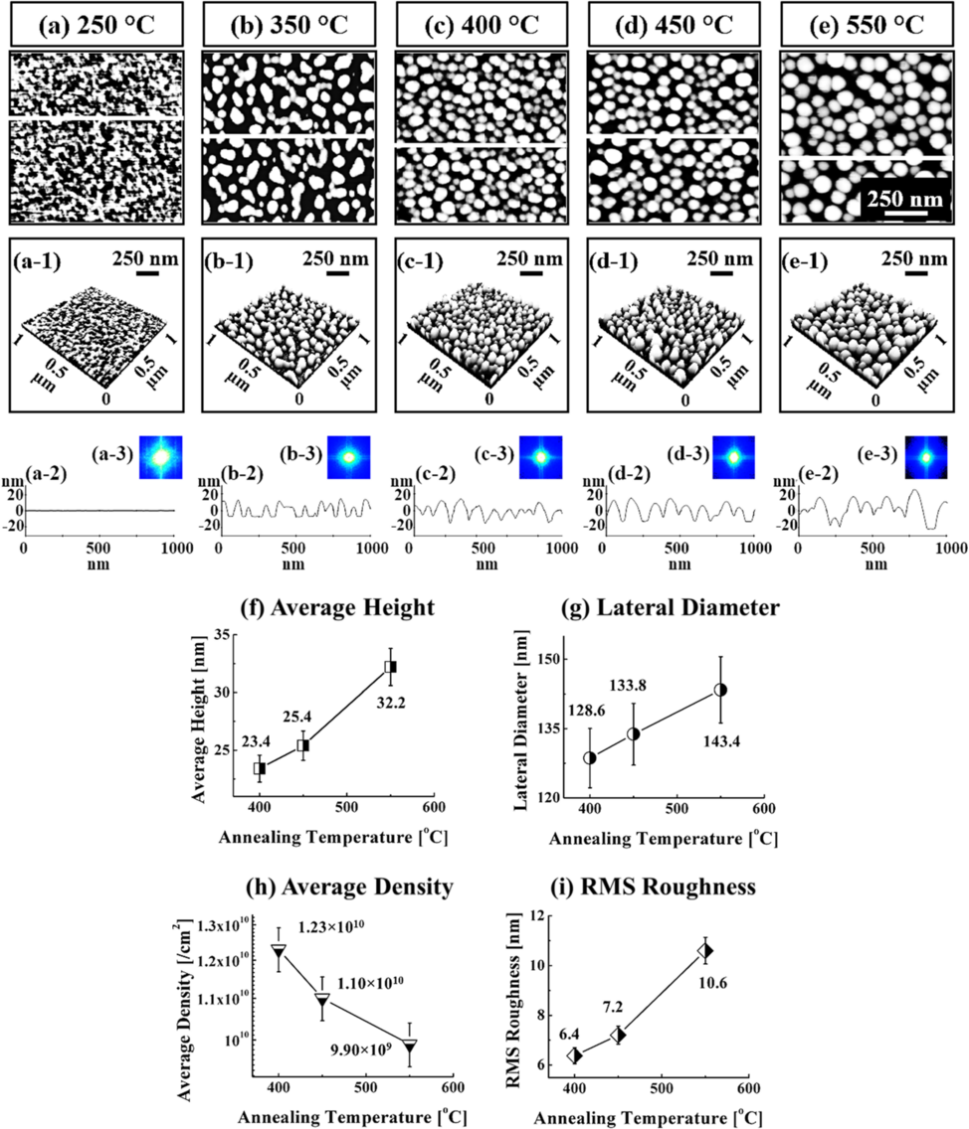
The evolution of self-assembled Au droplets induced by annealing temperature deviation between 250 and 550 °C on GaAs (111). **a**–**e** AFM top-views. (*a-1*)–(*e-1*) corresponding line-profiles, acquired from white lines drawn in (**a**–**e**). (*a-2*)–(*e-2*) 2-D FFT power spectra. **f**–**i** average height, lateral diameter, density, and RMS roughness. *Error bars* are ±5 %

**Fig. 6:**
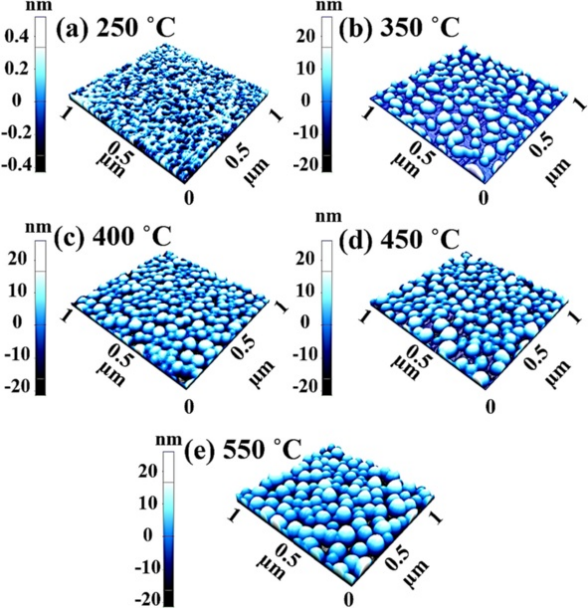
3-D AFM side views of the self-assembled Au droplets fabricated by the variation of the annealing temperature between 250 and 550 °C. The Au droplets were fabricated with the deposition of 2.5 nm for the annealing of 450 s. **a**–**e** AFM side views of 1 × 1 μm^2^

Figure 5 shows the evolution of Au droplets controlled by the AT between 250 and 550 °C on GaAs (111) at the fixed DT of 450 s with the DA of 2.5 nm. Figure 6 shows the corresponding 3-D AFM side views of the samples. Generally, depending on the AT, the distinctive surface morphology evolution was observed from the Au clusters to round-dome-shaped Au droplets as clearly shown in Figs. 4a–e and 5a–e. With the AT variation, two phases of evolution were observed: Au cluster phase below 400 °C and droplet phase above 400 °C. For example, at the AT of 250 °C, although the surface was still very smooth and even comparable to the bare surface as revealed in Fig. 4a-1, Au adatoms started to aggregate as shown in Figs. 4a and 5a. When the AT reached 350 °C, a sharp transition from the connected wiggly geometry to the Au cluster was clearly observed in Figs. 4b and 5b. Obviously, increased AT can provide enhanced thermal energy to the Au Adatoms, and thus, the Au agglomeration happened more drastically, resulting in the Au clusters with a limited diffusion [45]. The relationship between the diffusion length and substrate temperature (*T*_sub_) can be simply expressed with an equation: ${l}_{\mathrm{D}}=\sqrt{D\tau }$ where *l*D is the diffusion length, *D* is the diffusion coefficient, and τ is the diffusion time of adatoms. And also, *D* can be written as *D* ∝ *T*sub where the *T*sub is the substrate temperature. In this case, every other parameter is fixed and only the AT was varied, and thus, we can directly observe the AT effect. The *l*D_,_ is a direct function of the AT and being provided with the stronger binding energy among the Au adatoms (E_I_ > E_S_) and lower surface energy and high chemical potential at the nucleus sites, the enhanced *l*D can lead to the enlarged absorption boundary. Such that we can expect the increased dimension of Au droplets at an elevated AT along with the reduced density, which is a quite similar effect to the DA variation, namely the increased dimension associated with the decreased density with the increased DA. Similarly, at the increased AT of 400 °C, with the increased *l*D, the connected wiggly nanostructures gradually developed into Au droplets as shown in Figs. 4c and 5c and formed the round-dome shaped Au droplets at higher ATs as shown in Figs. 4d and e and 5d and e due to favorable diffusion. In terms of the size and density evolution due to the AT variation, Fig. 4f–h summarizes the results: the AH, LD, and AD. At 400 °C, the AH was 23.4 nm, the LD was 128.6 nm, and the AD was 1.23 × 10^10^/cm^2^. At 450 °C, the AH was increased to 25.4 nm by × 1.09 and the LD was jumped to 133.8 nm by × 1.04. In compensation, the AD was decreased to 1.11 × 10^10^/cm^2^ by × 1.12 at the same time. Finally, at the AT of 550 °C, the AH was increased to 32.2 nm by × 1.27 and the LD was increased to 143.4 nm by × 1.07. Meanwhile, the AD was decreased to 9.90 × 10^9^/cm^2^ by × 1.11. Meanwhile, with the gradual increase of dimensions of the self-assembled Au droplets as a function of the AT, the RMS surface roughness was accordingly increased from 6.4 to 10.6 nm as shown in Fig. 4i. Overall, under the fixed DA and DT, the increased AT resulted in the increased dimensions in both average height and diameter and the average density was gradually decreased as can be expected with the simple diffusion theory. Obviously, this type of size and density evolution based on the AT variation can also be witnessed with various metal nanoparticles on various semiconductors substrates [46–50].

**Fig. 7:**
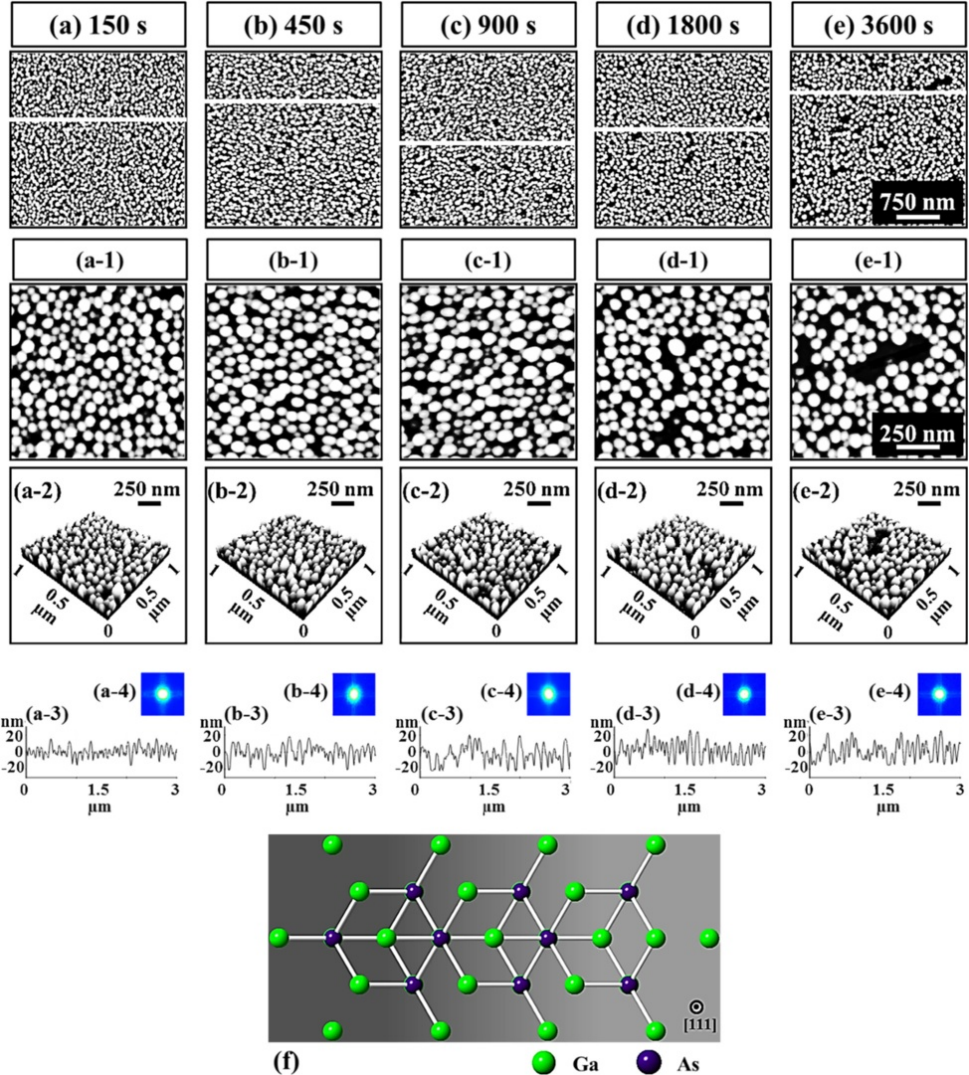
The dwelling time effect on self-assembled Au droplets. The dwelling time was varied from 150 to 3600 s on GaAs (111) at 550 °C. **a**–**e** AFM top views of 3 × 3 μm^2^. (*a-1*)–(*e-1*) AFM top views of 1 × 1 μm^2^. (*a-2*)–(*e-2*) cross-sectional line-profiles. (*a-3*)–(*e-3*) FFT power spectra. **f** Ball and stick model of GaAs (111)

**Fig. 8:**
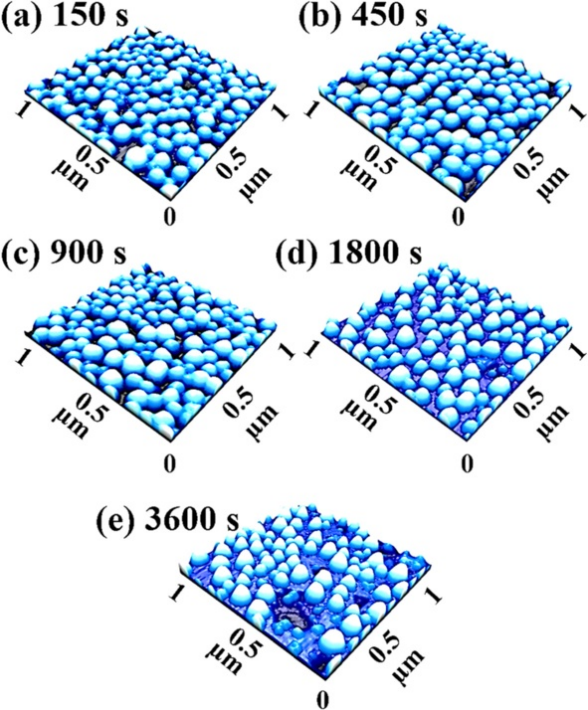
3-D AFM side views of the self-assembled Au droplets fabricated by the variation of the annealing time from 150 to 3600 s on GaAs (111). The Au droplets were fabricated with the deposition of 2.5 nm at 550 °C. **a**–**e** AFM side views of 1 × 1 μm^2^

**Fig. 9:**
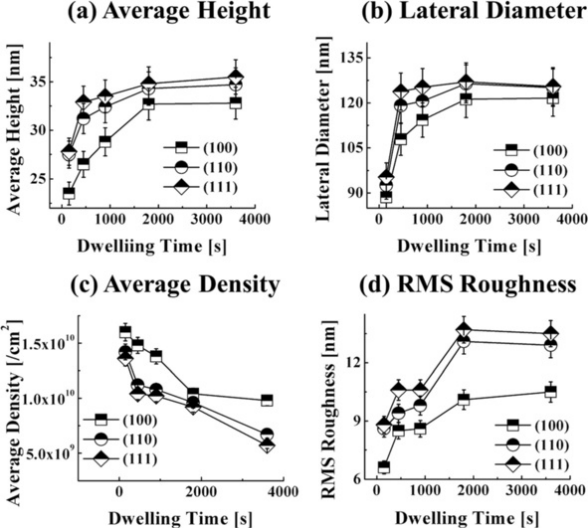
Plots of the average height (**a**), lateral diameter (**b**), average density (**c**), and the RMS surface roughness (**d**) of self-assembled Au droplets on various GaAs surfaces annealed for the dwelling time between 150 and 3600 s at 550 °C. *Error bars* are ±5 %

Figure 7 shows the DT variation effect on the self-assembled Au droplets fabricated on GaAs (111) with the fixed AT and DA of 550 °C and 2.5 nm, and the ball and stick model of GaAs (111) is shown in Fig. 7f. Figure 8 shows the 3-D AFM side views of the Au droplets on GaAs (111), and the 3-D AFM side views of GaAs (100) and (110) are supplied in Additional file 1: Figure S5 and Additional file 1: Figure S6. Generally, the self-assembled Au droplets gradually grew larger in terms of the average size, and correspondingly, the average density was consistently decreased with the increased DT as summarized and plotted in Table 1 and Fig. 9. For instance, with the DT of 150 s, relatively smaller round-dome-shaped Au droplets were resulted on GaAs (111) as shown in Figs. 7a, a-1 and 8a. As summarized in Table 1, the AH was 27.8 nm, the LD was 95.3 nm, and the average density was 1.36 × 10^10^/cm^2^. With the DT of 450 s, the size gradually increased as shown in Figs. 7b, b-1 and 8b: the AH increased to 32.9 nm by × 1.18 and the LD to 123.8 nm by × 1.30. Meanwhile, the AD decreased to 1.04 × 10^10^/cm^2^ by × 1.31. With the DT of 900 and 1800 s, again, the size gradually increased with correspondingly decreased density as in Figs. 7c-d, c-1, d-1 and 8c-d. The AH increased to 34.8 nm and the LD to 127.0 nm while the AD accordingly decreased to 9.20 × 10^9^/cm^2^. Lastly, with the DT of 3600 s, the size of Au droplets grew a little bigger in height and the surface showed large empty areas as shown in Figs. 7e, e-1 and 8e, which likely can be due to the desorption of As, resulting in the decrement of density. The AH was 35.5 nm and the LD was 125.5 nm and the AD significantly decreased to 5.70 × 10^9^/cm^2^ by × 1.61. Overall, along with the extended DT, the size of Au droplets gradually increased and the density decreased accordingly while the alteration on the Au droplets caused by the DT variation was somewhat less significant as compared to the DA variation. The DT-related Au droplet evolution can be related to the Ostwald ripening theory and according to the theory [39–41]. The mean radius of Au droplets < *R*_Au_ > is given as a function of the dwelling time *t*: <*R*Au(*t*) > ^*n*^ − <*R*_0_ > ^*n*^ = K*t [40, 41], where < *R*0 > is the radius of Au droplets at *t* = 0, *n* is the growth exponent depending on the growth mode: i.e., in 2-D case *n* = 3 and *n* = 4 for the case 3-D. Also, the K* is given as ${K}^{*}=\frac{8{N}_0D\gamma {\varOmega}^2}{45{k}_B Tl(L)}$ [40]. Considering this experimental condition, the density of nucleation sites (*N*0) is 1.22 × 10^15^/cm^2^, the interface energy (γ) between Au and air is 1.5 J/m^2^, the atomic volume of Au (Ω^2^) is 1.69 × 10^−29^ cm^3^, the T is an absolute temperature, and the characteristic length *l*(L) is ~3. Based on the theory, the radius of Au droplets is very strongly dependent on the DT as seen; however, the size increase is somewhat indefinite. The size evolution based on the Ostwald ripening [39–41] generally aggressively occurs at the relatively earlier stage of annealing; however, as time goes by, it reaches the saturation and gets slowed down as can be seen in these experiments.

**Table 1:** Summary of the average height, lateral diameter, density, and RMS surface roughness of the self-assembled Au droplets on various GaAs surfaces

	*I*	*T*_d_	150	450	900	1800	3600
Average height (AH) (nm)	(100)		23.5	26.5	28.8	32.7	32.8
	(110)		27.5	31.2	32.4	34.3	34.7
	(111)		27.8	32.9	33.5	34.8	35.5
Lateral diameter (LD) (nm)	(100)		88.6	107.9	114.3	121.2	121.6
	(110)		92.7	119.1	120.6	126.4	125.1
	(111)		95.3	123.8	125.2	127	125.5
Average density (AD) (×10^10^ cm^−2^)	(100)		1.6	1.48	1.38	1.04	0.98
	(110)		1.42	1.12	1.08	0.96	0.67
	(111)		1.36	1.04	1.02	0.92	0.57
RMS roughness (nm)	(100)		6.6	8.5	8.6	10.1	10.5
	(110)		8.6	9.4	9.8	13.1	12.9
	(111)		8.8	10.6	10.6	13.7	13.5

Samples were fabricated by the variation of dwelling time between 150 and 450 s at 550 °C. Data were obtained by the measurement of samples more than 100. The measurement error can be within ±5 %

*I* index of surfaces, *Td* dwelling time (s)

**Fig. 10:**
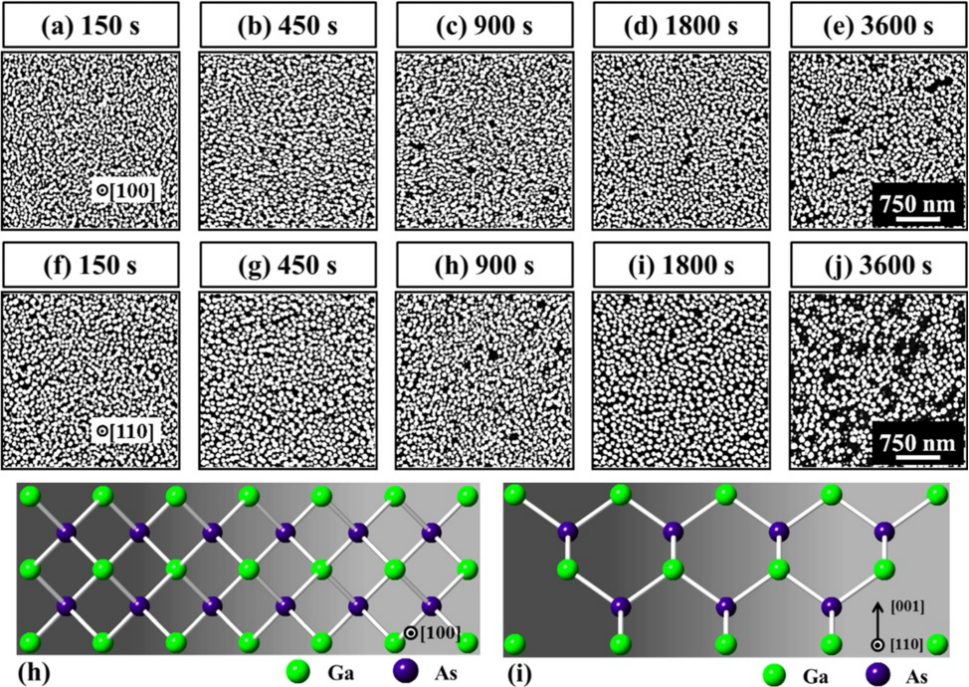
Self-assembled Au droplets fabricated by the variation of dwelling time on GaAs (100) and (110). AFM top views (3 × 3 μm^2^) of Au droplets on (100) in (**a**–**e**) and on (110) in (**f**–**j**). Ball and stick model of GaAs (100) in (**h**) and GaAs (110) in (**i**)

Similarly, Fig. 10 summarizes the results of the DT variation on GaAs (100) and (110) surfaces for the identical condition (fixed AT at 550 °C and DA of 2.5 nm) and the ball and stick model of (100) and (110) is shown in Fig. 10ah and i. Generally, similar to the Au droplets on (111), a gradual size increase with the decreased density was also observed on these two surfaces of GaAs (100) and (110) during the DT variation, whereas clear differences among the surfaces in terms of the size and density were constantly observed at the same time. The size of the self-assembled Au droplets was constantly smaller on (100) at each DT as well summarized in Fig. 9b and b, and the average density was the highest accordingly as shown in Fig. 9b. (111) surface showed the largest size out of the three, and correspondingly, the smallest in the average density, resulting in (100) < (110) < (111) in size and vice versa in density. The RMS roughness also showed a correlation with the size and density as well summarized in Fig. 9b. The diffusion length (*l*D) can be affected by the surface morphology, which can be caused by many factors including surface reconstruction, dangling bonds, step density, etc. [51]. The ideal surface energy of GaAs (100), (110), and (111) were 65, 57, and 62 meV/Å^2^, which can theoretically result in difference for diffusion length [51]. Higher surface energy can indicate a shorter *l*D if the other conditions were fixed. However, in this work, the Au droplets on GaAs (111) were always with the largest size and the lowest density, and the Au droplets on (100) consistently possessed the smallest size with the highest density as discussed. We note that the index-dependent size and density can be related to the different *l*D affected by the surface roughness [52–54], and to quantify this, the RMS roughness (*R*_q_) of the initial substrates was measured with the software-assisted calculations of the obtained data from AFM images. The measured *R*_q_ values were 0.226 nm for (100), 0.207 nm for (110), and 0.196 nm for (111), and accordingly, the *R*_q_ values were consistent with the results discussed here. As can be expected, the surface with a higher *R*_q_ can possess a relatively shorter diffusion length and thus can result in relatively smaller size of Au droplets with a lower density and vice versa.

## Conclusions

In summary, the control of self-assembled Au droplets on GaAs (111), (110), and (100) is systematically investigated through the variation of DA, AT, and DT in this paper. Resulting Au droplets and nanostructures were characterized and analyzed by the AFM images, cross-sectional line-profiles, FFT power spectra, EDS spectra, and size-density plots. Depending on the DA variation between 2 and 20 nm, the self-assembled Au droplets drastically evolved from the tiny-dense droplets to the super-large droplets and the evolution was explained based on the Volmer–Weber growth model. During the evolution, the AH significantly increased over 400 % from 23.1 to 96.5 nm and the LD was increased over 800 % from 52.5 to 432.9 nm, showing a preferential lateral growth. To compensate the size increase, the AD was sharply decreased from 4.23 × 10^10^/cm^2^ down to 1.16 × 10^8^/cm^2^. Depending on the AT variation between 250 and 550 °C, the radical evolution of surface morphologies was witnessed from the irregular wiggly nanostructures to the round-dome-shaped Au droplets. Throughout the AT range, the increased size was associated with the decreased density as a function of temperature and described by the relationship between the diffusion length and substrate temperature (*T*_sub_). Depending on the DT variation, the size increment of the Au droplets associated with the density decrement was equally observed on GaAs (111), (110), and (100) and the difference in terms of size and density within the DT range between 150 and 3600 s was clearly observed among the surfaces (GaAs (100) < (110) < (111) in size and vice versa in density), which was discussed in terms of the Ostwald ripening and diffusion length (*l*D) associated with the RMS roughness.

## Additional file

Additional file 1: Figure S1. Surface morphologies of bare (a) GaAs (100), (b) (110), and (c) (111) surfaces. Cross-sectional line-profiles in (a-1–c-1) are acquired from the white lines drawn in the corresponding AFM top-views. (a-2–c-2) 2-D FFT power spectra. As shown in (a–c), the surface appears very smooth for each surface, which was clearly evidenced with line-profiles in (a-1–c-1). **Figure S2.** Photoluminescence spectra of bare GaAs (100), (110), and (111) substrates measured at room temperature. The emission was excited with a CW diode-pumped solid-state (DPPS) laser of a wavelength of 532 ± 1 nm at the output power of 120 mW and measured by the TE cooled CCD detector. Peaks are at 893.1, 877.3, and 891.5 nm with the full width at half maximum (FWHM) of 50.6, 58.9, and 62 nm, respectively. **Figure S3.** Raman spectra of GaAs (100), (110), and (111) substrates, measured at room temperature. Excitation was with a laser of 532 nm. Due to the diamond structure, backscattering only resulted with longitudinal optical phonon (LO) peak at 290.6 cm^−1^ on (100). Similarly, only transverse optical phonon (TO) peak was observed at 264.7 cm^−1^ on (110) due to the lattice structure. Both the LO and TO peaks were observed on (111) at 266 and 290.6 cm^−1^, respectively. **Figure S4.** Surface morphologies with (a) 2, (b) 4, (c) 6, and (d) 20 nm thickness of Au deposition on GaAs (111) as examples and other surface showed similar behaviors. Line-profiles in (a-1–d-1) show the cross-sectional morphologies and the location of line-profiles are indicated with the white lines drawn in the corresponding AFM top-views. (a-2–d-2) 2-D FFT power spectra. (a-3–d-3) height distribution histograms around zero and got slightly wider as the deposition amount (DA) was increased from ±0.5 to ±2 nm. At each DA, the surfaces appeared smooth as witnessed by the cross-sectional line-profiles in (a-1–d-1). **Figure S5.** 3-D AFM side views of the self-assembled Au droplets on GaAs (100) fabricated by the variation of the annealing time from 150 to 3600 s. The Au droplets were fabricated with the deposition of 2.5 nm at 550 °C. (a–e) AFM side views of 1 × 1 μm^2^. **Figure S6.** 3-D AFM side views of the self-assembled Au droplets on GaAs (110) fabricated by the variation of the annealing time from 150 to 3600 s. The Au droplets were fabricated with the deposition of 2.5 nm at 550 °C. (a–e) AFM side views of 1 × 1 μm^2^.
