# Supplementary material for: Observation of Shape, Configuration, and Density of Au Nanoparticles on Various GaAs Surfaces via Deposition Amount, Annealing Temperature, and Dwelling Time
Source: Nanoscale Res Lett. 2015 May 27;10:240. doi: 10.1186/s11671-015-0950-z (PMC4452356; doi:10.1186/s11671-015-0950-z)
Supplement: Additional file 1: Figure S1. — Surface morphologies of bare (a) GaAs (100), (b) (110), and (c) (111) surfaces. Cross-sectional line-profiles in (a-1–c-1) are acquired from the white lines drawn in the corresponding AFM top-views. (a-2–c-2) 2-D FFT power spectra. As shown in (a–c), the surface appears very smooth for each surface, which was clearly evidenced with line-profiles in (a-1–c-1). Figure S2. Photoluminescence spectra of bare GaAs (100), (110), and (111) substrates measured at room temperature. The emission was excited with a CW diode-pumped solid-state (DPPS) laser of a wavelength of 532 ± 1 nm at the output power of 120 mW and measured by the TE cooled CCD detector. Peaks are at 893.1, 877.3, and 891.5 nm with the full width at half maximum (FWHM) of 50.6, 58.9, and 62 nm, respectively. Figure S3. Raman spectra of GaAs (100), (110), and (111) substrates, measured at room temperature. Excitation was with a laser of 532 nm. Due to the diamond structure, backscattering only resulted with longitudinal optical phonon (LO) peak at 290.6 cm−1 on (100). Similarly, only transverse optical phonon (TO) peak was observed at 264.7 cm−1 on (110) due to the lattice structure. Both the LO and TO peaks were observed on (111) at 266 and 290.6 cm−1, respectively. Figure S4. Surface morphologies with (a) 2, (b) 4, (c) 6, and (d) 20 nm thickness of Au deposition on GaAs (111) as examples and other surface showed similar behaviors. Line-profiles in (a-1–d-1) show the cross-sectional morphologies and the location of line-profiles are indicated with the white lines drawn in the corresponding AFM top-views. (a-2–d-2) 2-D FFT power spectra. (a-3–d-3) height distribution histograms around zero and got slightly wider as the deposition amount (DA) was increased from ±0.5 to ±2 nm. At each DA, the surfaces appeared smooth as witnessed by the cross-sectional line-profiles in (a-1–d-1). Figure S5. 3-D AFM side views of the self-assembled Au droplets on GaAs (100) fabricated by the variation of the annealing time from [file 11671_2015_950_MOESM1_ESM.docx]

**Supplementary materials**

***Observation of Shape, Configuration and Density of Au Nanoparticles on Various GaAs Surfaces* *via Deposition Amount, Annealing Temperature and Dwelling Time***

Daewoo Lee^1^, Ming-Yu Li^1^, Mao Sui^1^, Quanzhen Zhang^1^, Puran Pandey^1^, Eun-Soo Kim^1^ and Jihoon Lee^1,2*^

^1^ College of Electronics and Information, Kwangwoon University, Nowon-gu Seoul 139-701, South Korea

^2^ Institute of Nanoscale Science and Engineering, University of Arkansas, Fayetteville AR 72701, USA

**References**

1. G. Abstreiter, E. Bauser, A. Fischer, and K. Ploog, *Applied Physics*, **16**, 345-352(1978).

2. A. Mooradian and G. B. Wright, *Solid State Communications*, **4**, 431–434(1966).


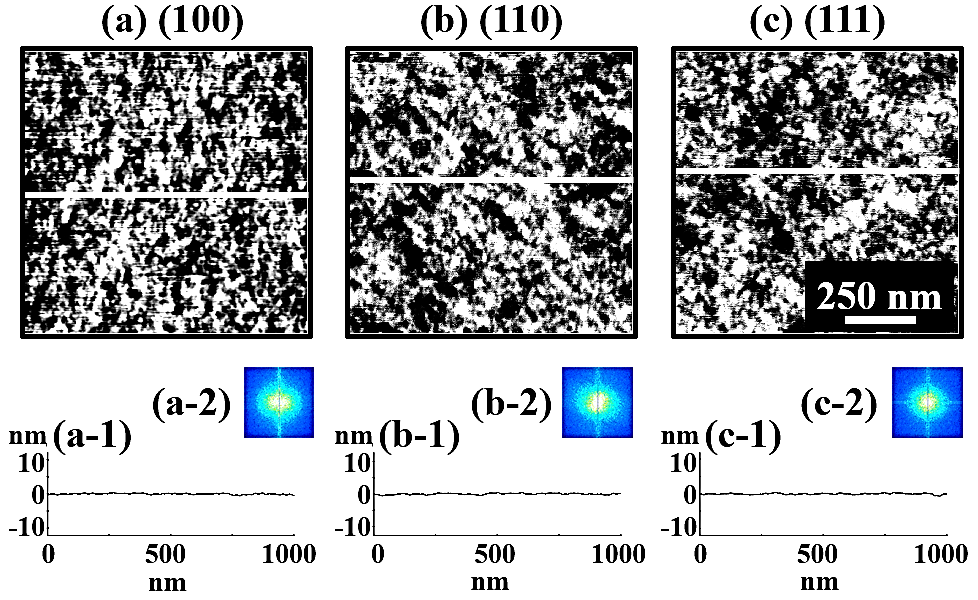


**Figure S1:** Surface morphologies of bare (a) GaAs (100), (b) (110) and (c) (111) surfaces. Cross-sectional line-profiles in (a-1) - (c-1) are acquired from the white lines drawn in the corresponding AFM top-views. (a-2) - (c-2) 2-D FFT power spectra. As shown in (a) - (c), the surface appear very smooth for each surfaces, which was clearly evidenced with line-profiles in (a-1) - (c-1).


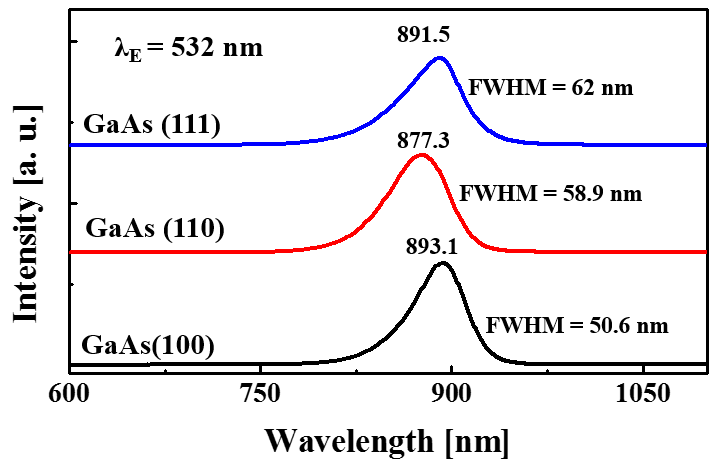


**Figure S2:** Photoluminescence spectra of bare GaAs (100), (110), and (111) substrates measured at the room temperature. The emission was excited with a CW diode-pumped solid-state (DPPS) laser of a wavelength of 532±1 nm at the output power of 120 mW and measured by the TE cooled CCD detector. Peaks are at 893.1, 877.3 and 891.5 nm with the full width at half maximum (FWHM) of 50.6, 58.9, and 62 nm respectively.


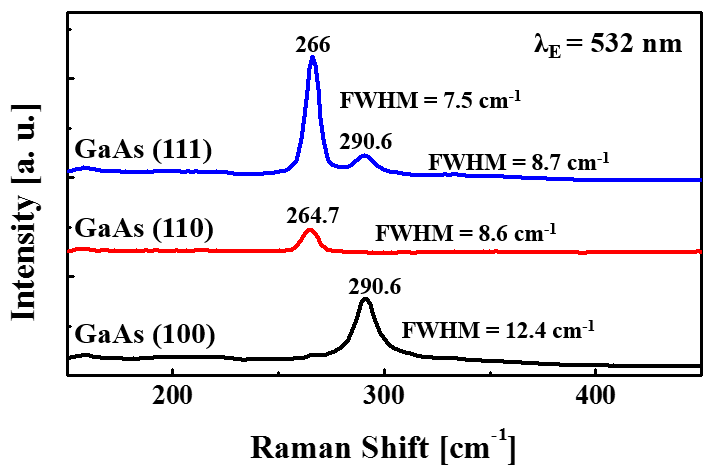


**Figure S3:** Raman spectra of GaAs (100), (110), and (111) substrates, measured at room temperature. Excitation was with a laser of 532 nm. Due to the diamond structure, backscattering only resulted with longitudinal optical phonon (LO) peak at 290.6 cm^-1^ on (100). Similarly, only transverse optical phonon (TO) peak was observed at 264.7 cm^-1^ on (110) due to the lattice structure. Both the LO and TO peaks were observed on (111) at 266 and 290.6 cm^-1^ respectively.^1,2^


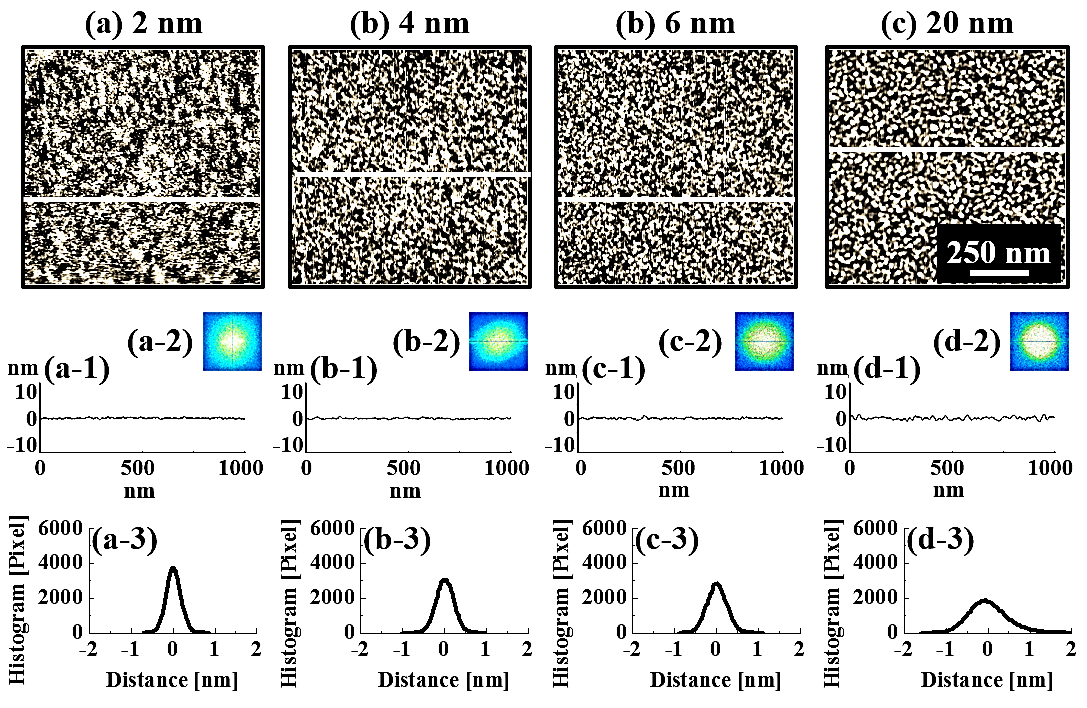


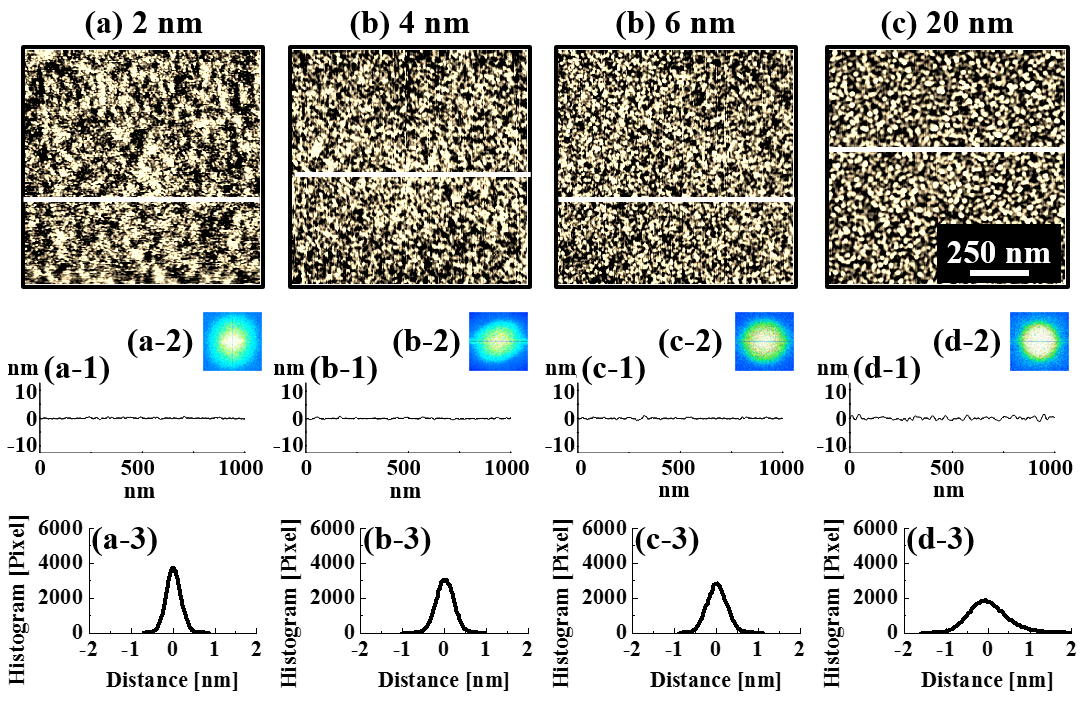


**Figure S4:** Surface morphologies with (a) 2, (b) 4, (c) 6 and (d) 20 nm thickness of Au deposition on GaAs (111) as examples and other surface showed similar behaviors. Line-profiles in (a-1) - (d-1) show the cross-sectional morphologies and the location of line-profiles are indicated with the white lines drawn in the corresponding AFM top-views. (a-2) - (d-2) 2-D FFT power spectra. (a-3) - (d-3) height distribution histograms around zero and got slightly wider as the deposition amount (DA) was increased from ± 0.5 to ± 2 nm. At each DA, the surfaces appeared smooth as witnessed by the cross-sectional line-profiles in (a-1) - (d-1).


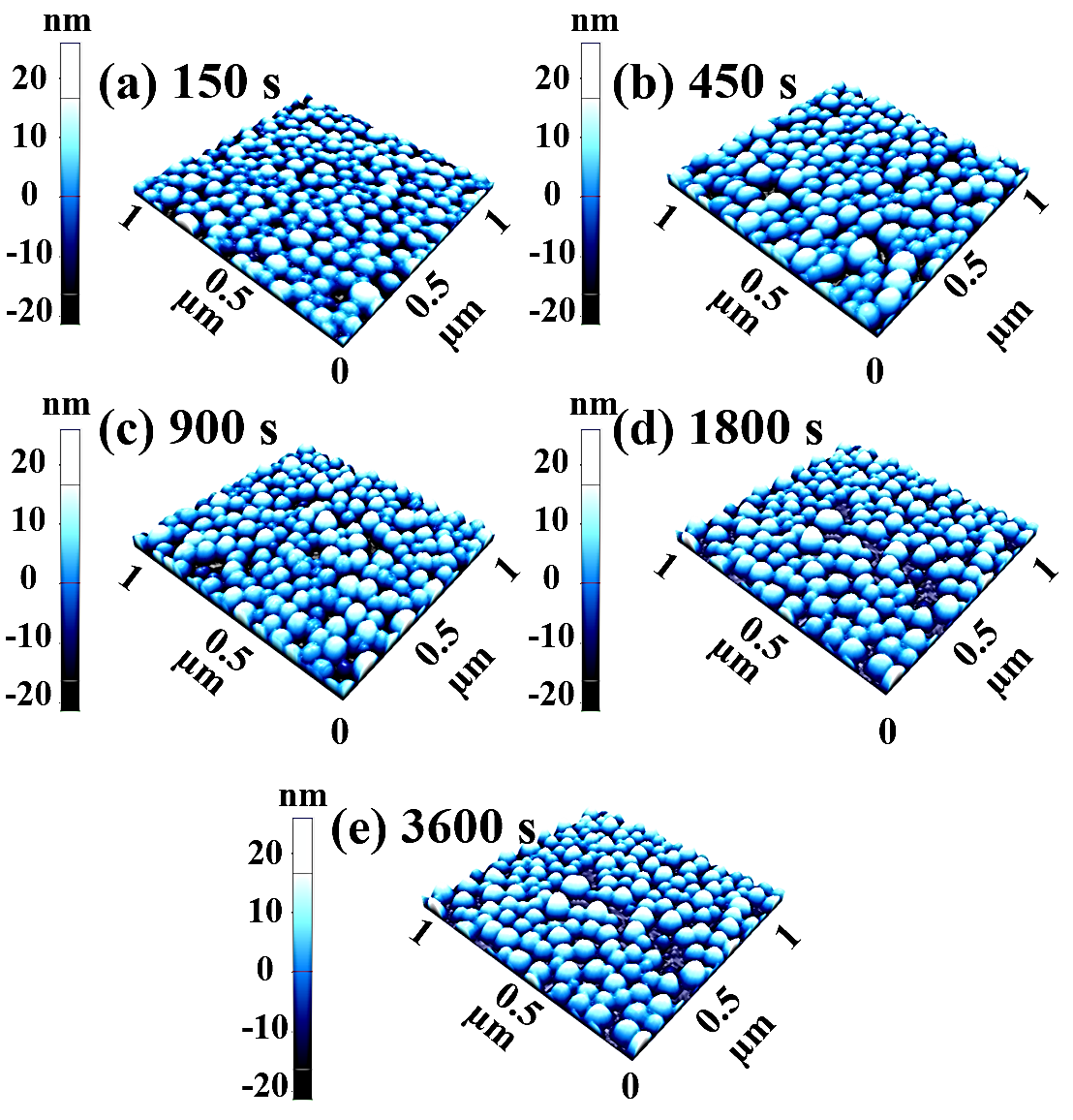


**Figure S5:** 3-D AFM side-views of the self-assembled Au droplets on GaAs (100) fabricated by the variation of the annealing time from 150 to 3600 s. The Au droplets were fabricated with the deposition of 2.5 nm at 550 ^o^C. (a) – (e) AFM side-vews of 1 × 1 µm^2^.


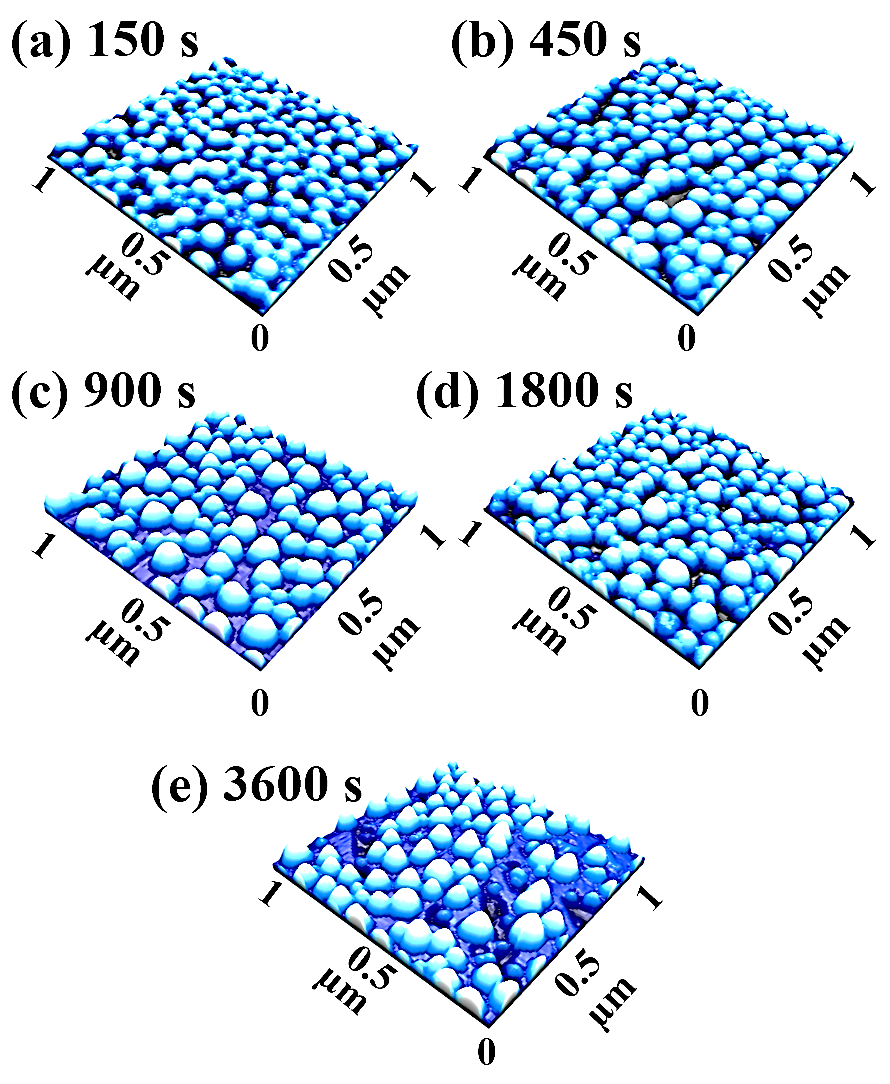


**Figure S6:** 3-D AFM side-views of the self-assembled Au droplets on GaAs (110) fabricated by the variation of the annealing time from 150 to 3600 s. The Au droplets were fabricated with the deposition of 2.5 nm at 550 ^o^C. (a) – (e) AFM side-vews of 1 × 1 µm^2^.
